# Synthesis of Tert-Octylsalicylaldoxime and Its Application in Extraction of Cu(II)

**DOI:** 10.3389/fchem.2021.592760

**Published:** 2021-08-13

**Authors:** Liqing Li, Luo Feng, Chunfa Liao, Fangxu Li, Liqin Yang

**Affiliations:** ^1^ Faculty of Materials Metallurgy and Chemistry, Jiangxi University of Science and Technology, Ganzhou, China; ^2^ Institute of Resources Comprehensive Utilization, Guangdong Academy of Science, Guangzhou, China

**Keywords:** tert-octylsalicylaldoxime, synthesis, characteristic, extraction performances, plating wastewater

## Abstract

The alkyl salicylaldoxime has attracted more and more attention recently due to the complex branched alkyl groups. In this study, a novel alkyl salicylaldoxime, tert-octylsalicylaldoxime, was successfully synthesized by the one-pot method. The yield and purity by the elemental analysis were 96.17 and 94.13%, respectively. The structure was confirmed by elemental analysis, FT-IR, ^1^H NMR (Nuclear Magnetic Resonance), ^13^C NMR spectroscopy, and MS. Results showed that tert-octylsalicylaldoxime with a new structure exhibited excellent extraction ability and selectivity for Cu(II) and can be successfully used to recover Cu from copper-nickel alloy electroplating wastewater. Thus, this product has the potential to be used as a powerful copper extractant in the future.

## Introduction

Because copper is one of the most important base metals, the big economic development year by year will cause a serious copper supply and demand contradiction (Yu et al., 2020). Although there are many ways to extract and recover copper ions, solvent extraction is still the most effective and key technique (Farrell et al., 2010; Watling et al., 2014; Edebali and Pehlivan, 2016; Wang et al., 2018; Elizalde et al., 2019; Wang et al., 2020). Therefore, it is urgent to improve the solvent extraction technology and develop novel extractants to overcome this contradiction (Li et al., 2011). In nearly one hundred years of hydrometallurgy history, the importance of chemical factors in achieving the targeted performance of extraction has been widely recognized. Alkyl salicylaldoxime series (AS), the N-O type chelating organic agents, is widely used as extractants to extract copper, nickel, zirconium, and molybdenum (Moradi Ali, 2012; Jain et al., 2016). Jiang et al. have investigated the performance of DZ988N (a mixture of equal volumes of 5-nonylsalicylaldoxime and 2-hydroxy-5-nonyl-acetophenone oxime) in separating copper from sulfate solution containing Cu^2+^, Fe^3+^, and Zn^2+^ (Jiang et al., 2018). Lasheen et al. have investigated the recovery of Mo(VI) from sulfate leach liquor containing Mo(VI) and U(VI) using 5-nonylsalicylaldoxime in a kerosene system (Lasheen et al., 2014). Sastre and Alguacil have used Lix 622 as an extractant to co-extract and selectively strip copper(II) (Sastre and Alguacil, 2001). Gameiro et al. have used Lix 84-I as an extractant to extract copper from the ammoniacal medium (Gameiro et al., 2010). 5-Nonylsalicylaldoxime (NSO) (Zhang et al., 2010), a kind of AS extractant, has been most widely used to extract Cu(II) as the main effective component in the common commercial extractants (see Table 1). In addition, minor changes to the chemical structure of the extracting molecule can have a very significant effect on the extracting agent’s performance in the extraction process.

**TABLE 1:** Alkyl salicylaldoxime investigated by some previous references.

Commercial name	Compounds	References
LIX622	2-Hydroxy-5-dodecyl salicylaldoxime, tridecyl alcohol	Alguacil and Cobo (1998)
LIX84	2-Hydroxy-5-mercaptoacetophenone oxime	Parija and Bhaskara Sarma (2000)
LIX984	2-Hydroxy-5-dodecyl salicylaldoxime, 2-hydroxy-5-mercaptoacetophenone oxime(1:1)	Aminian and Bazin (2000)
LIX984N	5-Nonylsalicylaldoxime, 2-hydroxy-5-mercaptoacetophenone oxime(1:1)	Zhang et al. (2010)
P50/M5640	5-Nonylsalicylaldoxime, nonylphenol	Yang et al. (2016)

The focus of this study was to create novel salicylaldoximes with new structures for Cu(II) extraction. Tert-octylsalicylaldoxime (TOSO) was successfully synthesized by the one-pot method in the laboratory (Li et al., 2017). Moreover, its properties and extraction ability for Cu(II) were investigated using the extraction test. This study may provide a promising extractant for the recovery and separation of Cu(II).

The NSO was chosen as a reference extractant. Because the structures of NSO and TOSO are very similar and belong to salicylic oxime, and NSO is the most successful copper commercial extractant.

## Experimental

### Materials and Reagents

All chemicals used in this work were of analytical grade, and all solutions at specified concentrations were prepared or diluted by deionized water. The stock solutions (0.1 mol/L) of Cu(II), Co(II), Fe(III), and Ni(II) were prepared from the sulfate salts in 1% H_2_SO_4_. The working solutions of metals were obtained by diluting these stock solutions before use. Salicylaldoxime (SA) solution was prepared by dissolving SA in kerosene. The pH values were adjusted by 20% (v/v) sodium hydroxide solution or sulfuric acid solution. The reagent was prepared daily.

The copper-nickel alloy electroplating wastewater was kindly supplied by Chengdu Quanrui Technology Co., Ltd. (Sichuan, China), which is produced by electroplating a copper-nickel alloy in a sulfate system. The wastewater mainly contains copper ions, nickel ions, and a small amount of electroplating additives. The copper content was 3.21 g/L, the nickel content was 1.52 g/L, and the pH was 1–2.

### Synthesis and Characterization of Tert-Octylsalicylaldoxime

#### Synthesis

**SCHEME 1:**
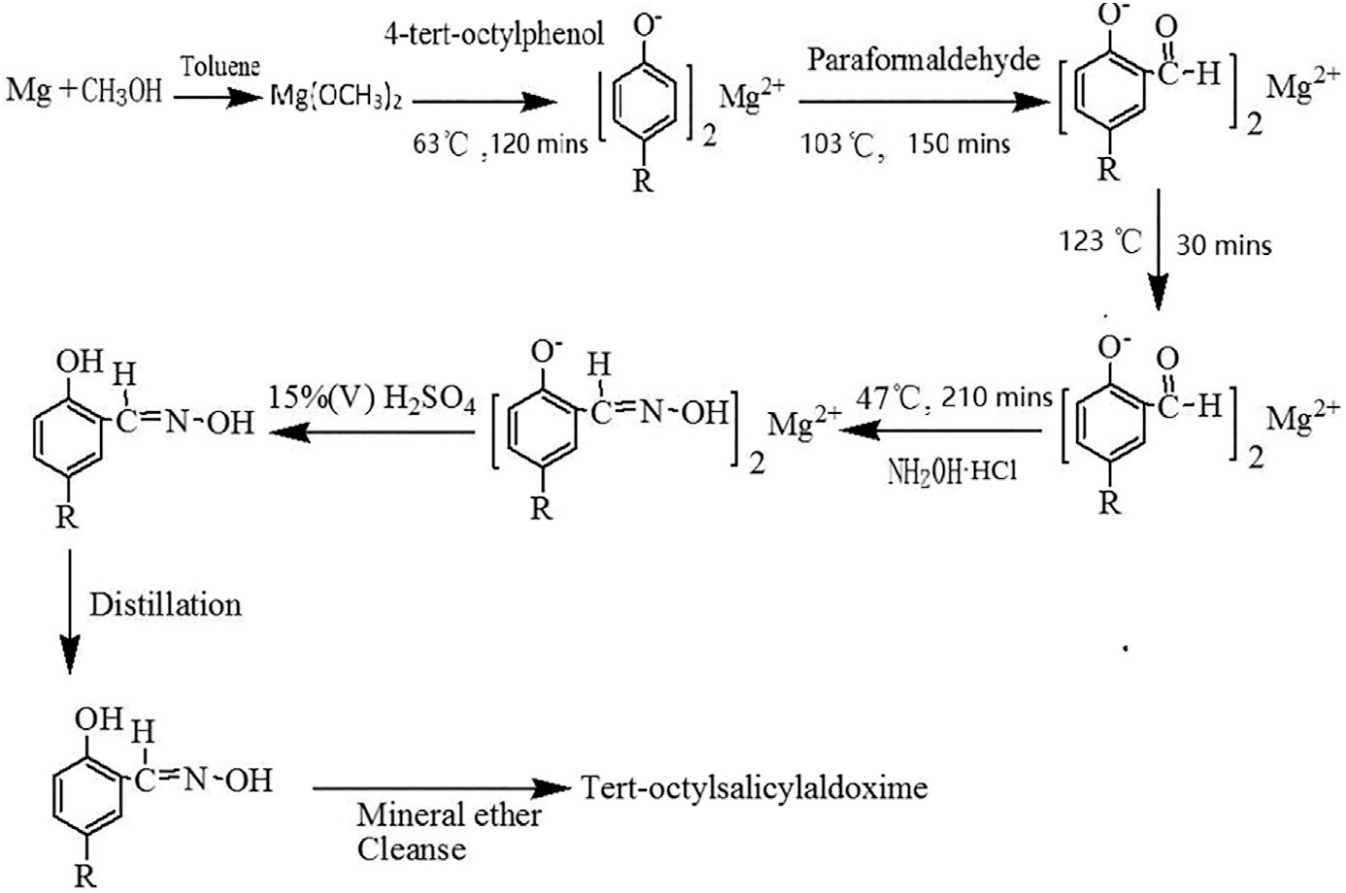
Synthesis of tert-octylsalicylaldoxime [R = –C(CH_3_)_2_CH_2_C(CH_3_)_3_].

The synthetic routes to TOSO are outlined in Scheme 1. Tert-octylsalicylaldoxime is mainly synthesized by magnesium, 4-tert-octylphenol, paraformaldehyde, and hydroxylamine hydrochloride. The optimized mole ratio of the four reactants is Mg:4-tert-octylphenol:paraformaldehyde:hydroxylamine hydrochloride = 0.55:1:2.40:1.50.

#### Formylation

Magnesium ribbons (0.022 mol) were added to the solution consisting of anhydrous methanol (50 ml) and toluene (30 ml), and then the mixture was heated to 63°C for reaction with stirring until all the magnesium was dissolved and H_2_ generation stopped. Then, 4-tert-octylphenol (8.25 g, 0.04 mol) was added to the mixture and refluxed for 120 min. Finally, the paraformaldehyde (0.096 mol) was added and then heated to 103°C for reaction with stirring for 150 min and subsequently heated to 123°C for reaction with stirring for 30 min.

#### Oximation

Firstly, the mixture was cooled to 47°C. Secondly, the hydroxylamine hydrochloride (0.06 mol) dissolved in deionized water was added to the above mixture and reacted for 210 min. Finally, tert-octylsalicylaldoxime (9.58 g, purity of 94.13%), appearing as white needle solid, was obtained after additional processes, including acidification, reduced pressure distillation, and petroleum ether cleaning.

#### Analytical Methods

The concentrations of C, H, and N of the final product were measured by Elementar-Vario elemental analyzer (Elementar Co., Germany). The purity and yield can be calculated by Eq 1 and Eq 2, respectively. Fourier Transform Infrared Spectrum (FT-IR) of the synthesized tert-octylsalicylaldoxime was surveyed from 400 to 4000 cm^−1^ by AVATR-360 FT-IR infrared spectrophotometer (Nicolet Co., United States) using KBr pellet technique. The ^1^H NMR (Nuclear Magnetic Resonance) and ^13^C NMR spectroscopy of the final product were obtained by ADVANCE III 500 NMR spectrometer (Bruker Co., Germany) using deuterium chloroform as solvent. The mass spectrometry was recorded by a GCMS-QP2010 analyzer (Japan) (Li et al., 2019; Wang et al., 2019).

$$\text{Purity}\left(\text{\%}\right)=\frac{\text{The}\;\text{measured}\;\text{concentration}\;\text{of}\;\text{N}}{\text{The}\;\text{theoretical}\;\text{concentration}\;\text{of}\;\text{N}}\times 100\%.$$ (1)

$$\text{Yield}\left(\text{\%}\right)=\frac{\text{The}\;\text{real}\;\text{product}\;\text{weight}}{\text{The}\;\text{theoretical}\;\text{product}\;\text{weight}}\times \mathrm{100\%}.$$ (2)

### Solvent Extraction Experiments

All the extraction and stripping experiments were conducted in a programmable air bath shaker with the organic phase (volume ratio W/O = 1). The stirring speed was maintained at 300 rpm with a stirring time ranging from 1 to 5 min to obtain extraction equilibrium. Initial pH was adjusted in the range of 1.0–3.5 with sulfuric acid or sodium hydroxide. The organic phase (extract) was stripped with sulfuric acid at a volume ratio W/O = 1. Unless otherwise stated, all other experiments were carried out at room temperature. A small amount of the raffinate (1 ml) was drawn out and diluted to the appropriate concentration for analysis.

Effects of extraction time, equilibrium pH, volume content, and A:O were investigated. The extraction efficiencies (E) of Cu(II) can be calculated from the differences between the concentrations of Cu(II) in the aqueous phase before and after extraction, as expressed by Eq. 3. The separation factor of the extractants for copper(II) to one metal (β_Cu/metal_) is evaluated by Eq. 4.

$$E=\frac{C_{1}V_{1}-C_{2}V_{2}}{C_{1}V_{1}}\times 100\%;$$ (3)

$${\text{β}}_{\text{Cu}/\text{metal}}=\frac{{\text{C}}_{\text{Cu}\left(\text{O}\right)}•{\text{C}}_{\text{metal}\left(\text{A}\right)}}{{\text{C}}_{\text{Cu}\left(\text{A}\right)}•{\text{C}}_{\text{metal}\left(\text{O}\right)}},$$ (4)

where E is the metal extraction efficiency, C_1_ and C_2_ are the metal concentrations in the aqueous phase before and after extraction, V_1_ and V_2_ are the volumes of the aqueous phase before and after the extraction; C_Cu(O)_ and C_Cu(A)_ indicate the concentration of copper(II) in the organic phase and aqueous phase (g/L), respectively, and C_metal(O)_ and C_metal(A)_ indicate the concentration of organic phase and aqueous phase (g/L), respectively. The concentration of metals in an aqueous solution is analyzed by Inductively Coupled Plasma-Atomic Emission Spectrometry (ICP-AES).

## Results and Discussion

### Characterization of Tert-Octylsalicylaldoxime

Chemical spectral data of TOSO were demonstrated as follows.

Element analysis results (%): (1) calculated, C 61.14, H 9.55, N8.92; (2) found, C 61.02, H 9.48, N 8.72. IR (KBr, cm^−1^): 3400 (OH), 2960 (CH_3_, CH_2_), 2800 (CH_3_, CH_2_), 1630(C=N), 1600 (aromatic CH), 1500 (aromatic CH), 1580 (aromatic CH), 1350 (NO).^1^H NMR (CDCl_3_, ppm): = 0.719 (t, 3H, J = 6.0 Hz, CH_3_), 1.343 (t,3H, J = 6.0 Hz, CH_3_), 1.701 (s, 1H, CH_2_), 7.13 (s, 1H, CH), 6.90 (d, 1H, CH),7.28 (d, 1H, J = 6.4 Hz, CH), 7.50 (t, 1H, J = 6.0 Hz, CH), 9.646–9.668 (s, 1H, OH), 8.233 (s, 1H, J = 6.4 Hz, OH).^13^C NMR (CDCl_3_, ppm): = 31.56, 31.79, 32.33, 56.89, 37.88,115.46, 129.47, 128.15, 141.47, 116.05, 154.88 and 153.62. MS for C_15_H_23_NO_2_ (M^+^): 249.35. Found: 249.

### Extraction and Stripping of Cu(II) Ion

**FIGURE 1:**
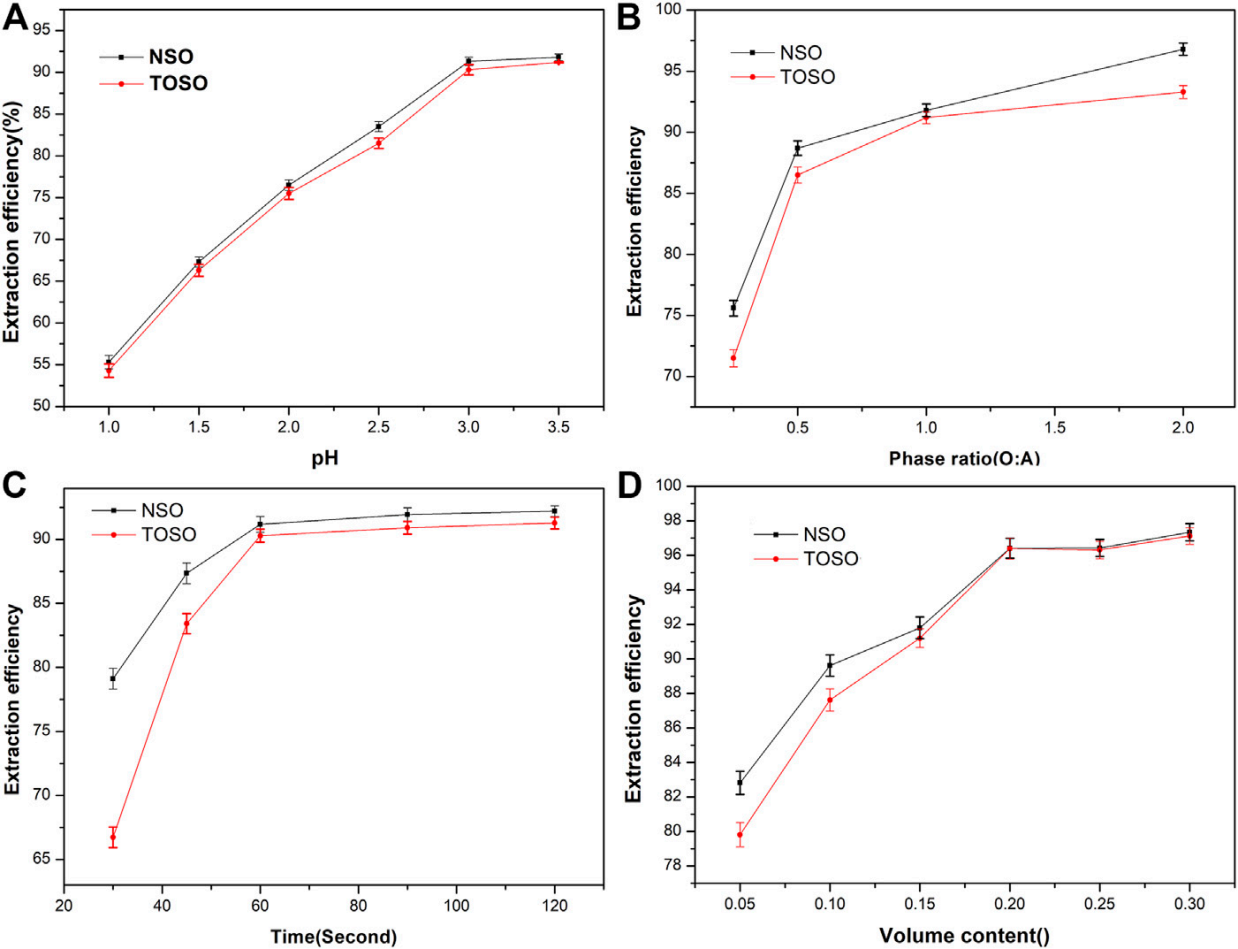
Effects of equilibrium pH **(A)**, phase ratio A:O **(B)**, extraction time **(C)**, and volume content **(D)** on the recovery of Cu(II) with TOSO and NSO.

Effects of extraction time, equilibrium pH, volume content, and A:O on extraction efficiency are shown in Figure 1. Those experiments were carried out in H_2_SO_4_ media with an initial Cu(II) concentration of 1.92 g L^−1^, 15% (V/V) of extractants, and the phase ratio (aqueous phase to organic phase) of 1 and were single-stage.

As can be seen in Figure 1A, extraction efficiency increased with pH under the experimental pH 1.0–3.5. At pH 3.0, TOSO and NSO extracted out 90.3 and 91.3% of Cu(II) from the aqueous phase, respectively. Figure 1B indicates that with the increase of phase ratio, TOSO and NSO exhibited more satisfactory extraction efficiency for Cu(II). When the phase ratios (O:A) were 0.5, 1, and 2, the copper extraction efficiencies of TOSO were 86.53, 91.20, and 94.32%, respectively, and those NSO were 88.71, 91.80, and 96.82%, respectively. It indicated that the extraction efficiency increased when the phase ratio (O:A) increased from 1 to 2. However, the operation with a higher O:A ratio has a potential risk of phase separation. Therefore, it is reasonable to use a phase ratio of 1 in the experiment. Figure 1C shows that TOSO and NSO have a similar tendency of extraction efficiency within a fixed time; more than 90% Cu(II) was recovered in 60 s. In addition, TOSO and NSO revealed similar extraction efficiency in high extractant concentrations, as shown in Figure 1D.

**FIGURE 2:**
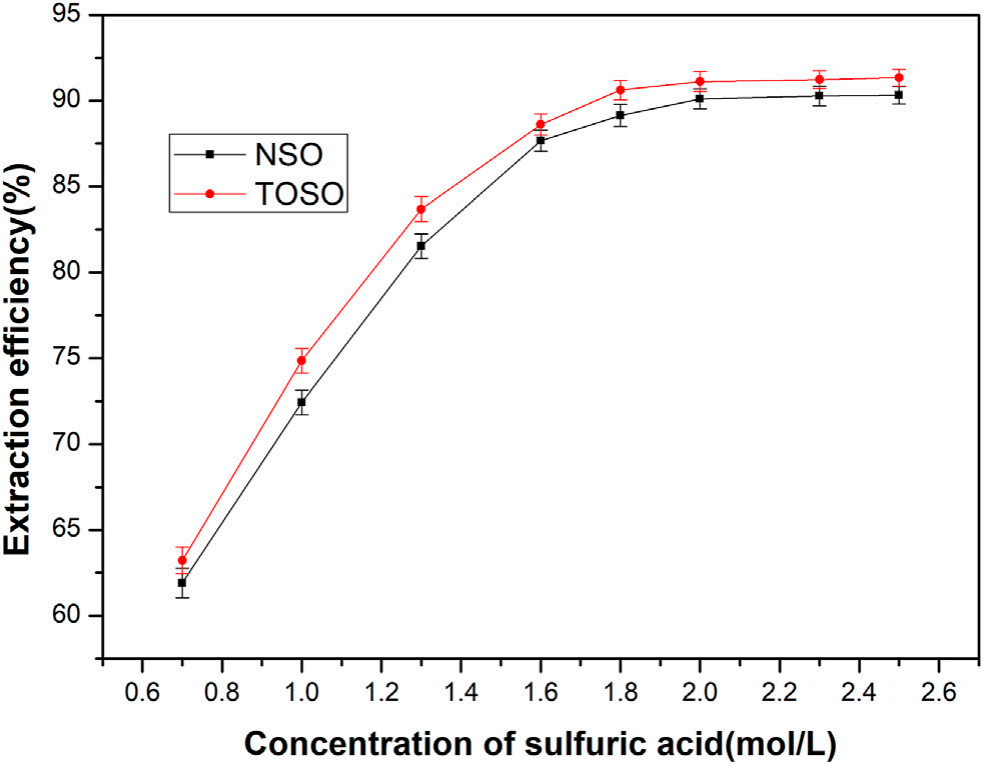
Influence of H_2_SO_4_ on Cu^2+^ stripping efficiency.

The copper-loaded organic was stripped at a 1:1 phase ratio with H_2_SO_4_ in a single stage. Results of these tests are listed in Figure 2. It is clear that the stripping efficiency of TOSO and NSO increased rapidly with the concentration of sulfuric acid and reached around 90% at the concentration of 1.8 mol/L. Further increase of sulfuric acid concentration did not have a significant effect on the stripping efficiency. Meanwhile, TOSO delivered better results than NSO in terms of stripping efficiency.

### Copper(II) Selectivity

In industrial applications, there are many different types of metal ions present in a single hydrometallurgical process, which can significantly interfere with the extraction of Cu(II). Therefore, it is necessary to investigate the selectivity of extractants for metals.

The extraction experiments were conducted in H_2_SO_4_ media with a phase ratio (O:A) of 1. A single-stage extraction was applied with an extraction time of 3 min at room temperature. The pH of the solution was adjusted by 20% (v/v) sodium hydroxide solution or sulfuric acid solution. The concentration of Cu^2+^ was 0.03 mol/L, while the concentrations of Fe^3+^, Zn^2+^, Ni^2+^, and Co^2+^ were set as 0.02 mol/L.

**FIGURE 3:**
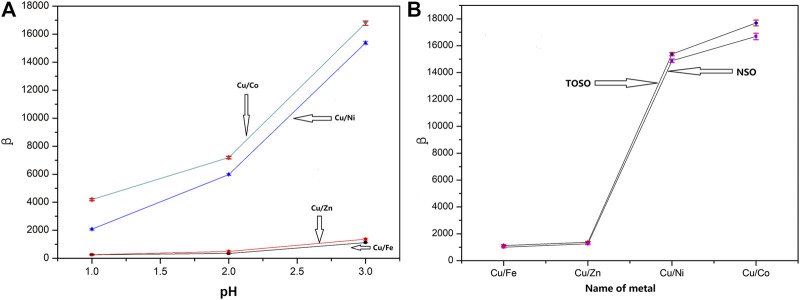
Selectivity of TOSO and NSO for Cu^2+^/metal ion in H_2_SO_4_ medium. **(A)** Selectivity of TOSO; **(B)** selectivity of TOSO and NSO at pH 3.

The experimental results are shown in Figure 3. As shown in Figure 3A, the synthesized tert-octylsalicylaldoxime presents a good extraction selectivity of Cu from other metals in the H_2_SO_4_ medium. The selectivity increased with the increase of pH from 1 to 3. The separation factor can be ranked in the following order: β_Cu/Co_ > β_Cu/Ni_ > β_Cu/Zn_ ≈ β_Cu/Fe_ within the pH studied, indicating that TOSO can selectively extract Cu from the complex solution. The strong selectivity for Cu may be caused by the particular umbrella-like structure of TOSO, which can provide a stronger steric effect than that of other AS series extractants with straight-chain R groups.

As can been seen in Figure 3B, when pH was 3.0, the β_Cu/Fe_, β_Cu/Zn_, β_Cu/Ni_, and β_Cu/Co_ values of TOSO in sulfuric acid are 1128, 1368, 15,375, and 16,787, respectively; the β_Cu/Fe_, β_Cu/Zn_, β_Cu/Ni_, and β_Cu/Co_ values of NSO are 1009, 1251, 14,875, and 15,867, respectively. Compared with NSO, TOSO showed better selectivity for Cu/metal separation.

### Tert-Octylsalicylaldoxime Application in Recovery of Cu(II) From Plating Wastewater

Considering the excellent separation performance of TOSO in Cu/Ni system, TOSO was selected for recovering Cu(II) from copper-nickel alloy electroplating wastewater. The experimental results, including extraction and stripping efficiency, extraction and stripping time, and β_Cu/Ni_ for all the copper extraction experiments, are summarized in Table 2.

**TABLE 2:** The experimental results of plating wastewater with Cu and Ni.

Name	Max extraction efficiency (%)	Max stripping efficiency (%)	Extraction separation time (s)	Stripping separation time (s)	β_Cu/Ni_
TOSO	88.42	85.61	42	45	131
NSO	89.21	84.83	35	39	101

The maximum extraction, maximum stripping efficiency, extraction separation time, stripping separation time, and separation factor of TOSO were 88.42%, 85.61%, 42 s, 45 s, and 131, respectively. Moreover, those of NSO were 89.21%, 84.83%, 35 s, 39 s, and 101, respectively. It indicated that NSO exhibited better performance in extraction efficiency and extraction and stripping phase separation time. However, TOSO showed advantages in separation factor and stripping efficiency.

Based on the above results, NSO has higher extraction ability and shorter phase separation time for Cu(II) than TOSO, while TOSO is superior to NSO in terms of the separation ability for metals (Ni, Co, Cu, Zn) and stripping efficiency. The alkyl side chains link of TOSO (C_8_) is shorter than that of NSO (C_9_), and the molecular sizes and Log P (Represents the hydrophobicity value) of TOSO are also smaller than those of NSO. This means that TOSO has a lower probability of capturing Cu(II) ions and weaker hydrophobicity, which results in a longer phase separation time. Compared with NSO, the better extraction selectivity of TOSO might be due to the particular umbrella-like structure of the tert-octyl group and shorter carbon chain (Mowafy and Mohamed, 2014).

## Conclusion

In this article, the extraction behavior of tert-octylsalicylaldoxime (TOSO) for Cu has been investigated by extraction and stripping tests. Based on the experimental results, the following conclusions could be drawn:

1) TOSO, a novel AS series extractant for Cu(II), was successfully synthesized by a one-pot reaction with a yield of 96.17% and purity of 94.13%. The structure of the synthesized TOSO was verified by elemental analysis, FT-IR, ^1^H NMR, and ^13^C NMR spectroscopy.
2) The results showed that TOSO exhibited powerful extraction and stripping performance. Compared with 5-nonylsalicylaldoxime (NSO), tert-octylsalicylaldoxime (TOSO) presents a stronger affinity to Cu(II) than to Co, Ni, Zn, and Fe in H_2_SO_4_ media, indicating it is suitable to extract Cu from these complex solutions selectively. Moreover, TOSO has been proved effective as a special extractant in the copper-nickel alloy electroplating wastewater. Therefore, TOSO can be considered a powerful extractant candidate for copper extraction in the hydrometallurgical process, which is of great significance to the development and utilization of copper resources.

## Data Availability

The original contributions presented in the study are included in the article/supplementary material; further inquiries can be directed to the corresponding authors.
